# Citizen Science Reveals an Extensive Shift in the Winter Distribution of Migratory Western Grebes

**DOI:** 10.1371/journal.pone.0065408

**Published:** 2013-06-19

**Authors:** Scott Wilson, Eric M. Anderson, Amy S. G. Wilson, Douglas F. Bertram, Peter Arcese

**Affiliations:** 1 Environment Canada, Canadian Wildlife Service, Saskatoon, Saskatchewan, Canada; 2 Centre for Wildlife Ecology, Simon Fraser University, Burnaby, British Columbia, Canada; 3 Department of Forest Sciences, University of British Columbia, Vancouver, British Columbia, Canada; 4 Environment Canada, Science and Technology Branch, c/o Institute of Ocean Sciences, Sidney, Canada; University of Alberta, Canada

## Abstract

Marine waterbirds have shown variable trends in abundance over the past four decades with some species displaying steep declines along the Pacific coast from British Columbia through California. One of the most dramatic changes has been that of western grebes (*Aechmophorus occidentalis*) in the Salish Sea. This region was a former core of the species wintering distribution but they have become increasingly rare prompting calls for conservation action. A more thorough understanding of this situation requires the analysis of trends at broader geographic scales as well as a consideration of mechanisms that might have led to a change in abundance. We used hierarchical modeling with a Bayesian framework applied to 36 years of Audubon Christmas Bird Count data to assess continent-wide and regional population trends in western and Clark’s grebes (*A. clarkii*) from 1975 to 2010. Our results show that the North American wintering population of *Aechmophorus* grebes decreased by ∼52% after 1975, but also that western grebes displayed strongly opposing regional patterns. Abundance decreased by about 95% over 36 years in the Salish Sea but increased by over 300% along coastal California. As a result, the mean centre of the species distribution shifted south by an estimated 895 km between 1980 and 2010. Mechanisms underlying this shift require further study but we hypothesize that it may be related to a change in the abundance and availability of their forage fish prey base. Since the mid-1980s, the Pacific sardine stock off the California coast increased from a few thousand metric tonnes to over two million. At the same time both the abundance and availability of Pacific herring declined in the Salish Sea. Studies are needed to examine this hypothesis further and additional consideration should be directed at other changes in the marine environment that may have contributed to a range shift.

## Introduction

Estimating population trends and identifying mechanisms underlying them are key tasks of population and conservation biologists [1]. Much of our knowledge about population ecology has been gained by focusing on species at single study sites, typically during the breeding period (reviewed in [2]). In contrast, citizen science data has facilitated analyses of ecological processes operating at broad spatial scales, including the environmental conditions that shape species distributions [3] and the influence of disease and climate on regional population dynamics [4], [5], [6]. A key advantage of such studies is to extend the spatial and temporal scope of analyses far beyond the limits of traditional field studies [7]. The incorporation of hierarchical models under a Bayesian framework have provided a further advantage by more precisely estimating population trends and the factors influencing them while accounting for the sources of variance typical in citizen science data [5], [8]. In this study, we used a hierarchical Bayesian approach applied to 36 yrs of Audubon Christmas Bird Count (CBC) data to examine the decline of western grebes (*Aechmophorus occidentalis*) in the former core of their non-breeding range in the Salish Sea of British Columbia and Washington state [9].

Marine birds along the North American Pacific Coast have shown variable patterns in abundance over the last few decades. Several piscivorous species have declined regionally in the California Current ecosystem [10], [11], [12] and in the Salish Sea of southern British Columbia and northern Washington [9], [13], but have increased further north [11], (but see [14]). The causes of regional population change are poorly understood but marine birds generally are affected by a suite of anthropogenic and natural factors. Many species are susceptible to by-catch in long-line and gillnet fisheries [15], pollution [14], [16], and the overharvesting of forage fish stocks [17]. Marine birds are also affected by oceanic regime shifts; periodic changes in sea-surface temperature, thermocline and degree of upwelling among regions that result in a cascading effect on nutrient transfer across trophic levels [18], [19]. The effects of regime shifts are particularly evident in highly productive regions, such as the California, Humboldt and Benguela Current ecosystems [20], [21], [22]. Fluctuations in ocean climate can directly influence reproduction and survival of marine birds through effects on prey availability [23], [24], but if sustained can also result in broader changes in distribution as they track spatial shifts in the location of key prey (e.g. [22], [25]).

Local surveys in the Salish Sea of British Columbia and Washington State have noted a dramatic decrease in abundance for western grebes over the past two to three decades [9], [13]. These declines, combined with perceived threats to some breeding colonies, have prompted calls for endangered species status in Canada (COSEWIC), British Columbia and Washington State. Western grebes are already listed as ‘sensitive’ in Alberta [26], which has historically supported the largest number of breeders among Canadian provinces. Due to these concerns, there is a need to evaluate population change in western and Clark’s grebes across the entire range of both species to provide a broader insight into local declines in the Salish Sea. Our specific aims in this study were to examine 1) continental patterns of population change, 2) regional variation in population trends that might be indicative of a shift in the non-breeding distribution and 3) what changes in the oceanic environment might have led to regional patterns of population change.

## Methods

### Western vs. Clark’s Grebes

A range-wide analysis of western grebe abundance is complicated by the relatively recent systematic designation of this species from its congener, the Clark’s grebe (*Aechmophorus clarkii*, [27]). The two species were considered to be color phases of a single species prior to 1985 and thus both recorded as western grebes on counts. Most grebes are identified to species on counts conducted after 1985 but because they are similar in appearance, positive identification is not always possible in large groups or at great distance. As a result, 13.9% of surveys after 1985 report ‘*Aechmophorus spp’* as a component of the total number of grebes recorded. In this case, we used the ratio of all western and Clark’s grebes identified on that count to estimate the fraction of *Aechmophorus* count attributed to each species. Clark’s grebes are rare compared to western grebes and only regularly detected in the southern portion of the *Aechmophorus* winter range. Thereore, our main analysis estimated trends for *Aechmophorus spp*. over the entire range under the assumption that large trends would necessarily be dominated by changes in the number of western grebes. We also conducted separate analyses for Clark’s grebes after 1985 to test for a potential influence on overall trends.

### Species Characteristics

*Aechmophorus* grebes breed in colonies at the vegetated edge of larger lakes and freshwater marshes. Kushlan et al. [28] estimated the North American population size of western grebes at more than 110,000 breeders, and Clark’s grebes at 10,000–20,000. The breeding range of western grebes includes most of southwest Canada and the western United States, while Clark’s grebes are more restricted to the southwestern states [27]. After breeding, western grebes migrate primarily to the Pacific coast and overwinter from southern Alaska to Mexico with lower numbers reported at inland sites from southern British Columbia to west Texas. Wintering Clark’s grebes are restricted to the southern portion of the western grebe winter range and are rare north of about 42° latitude (this study). Both species specialize on fish prey (>81% of diet) but are opportunistic and utilize other prey depending on availability [27].

### Assignment of Christmas Bird Count Data

**Figure 1:**
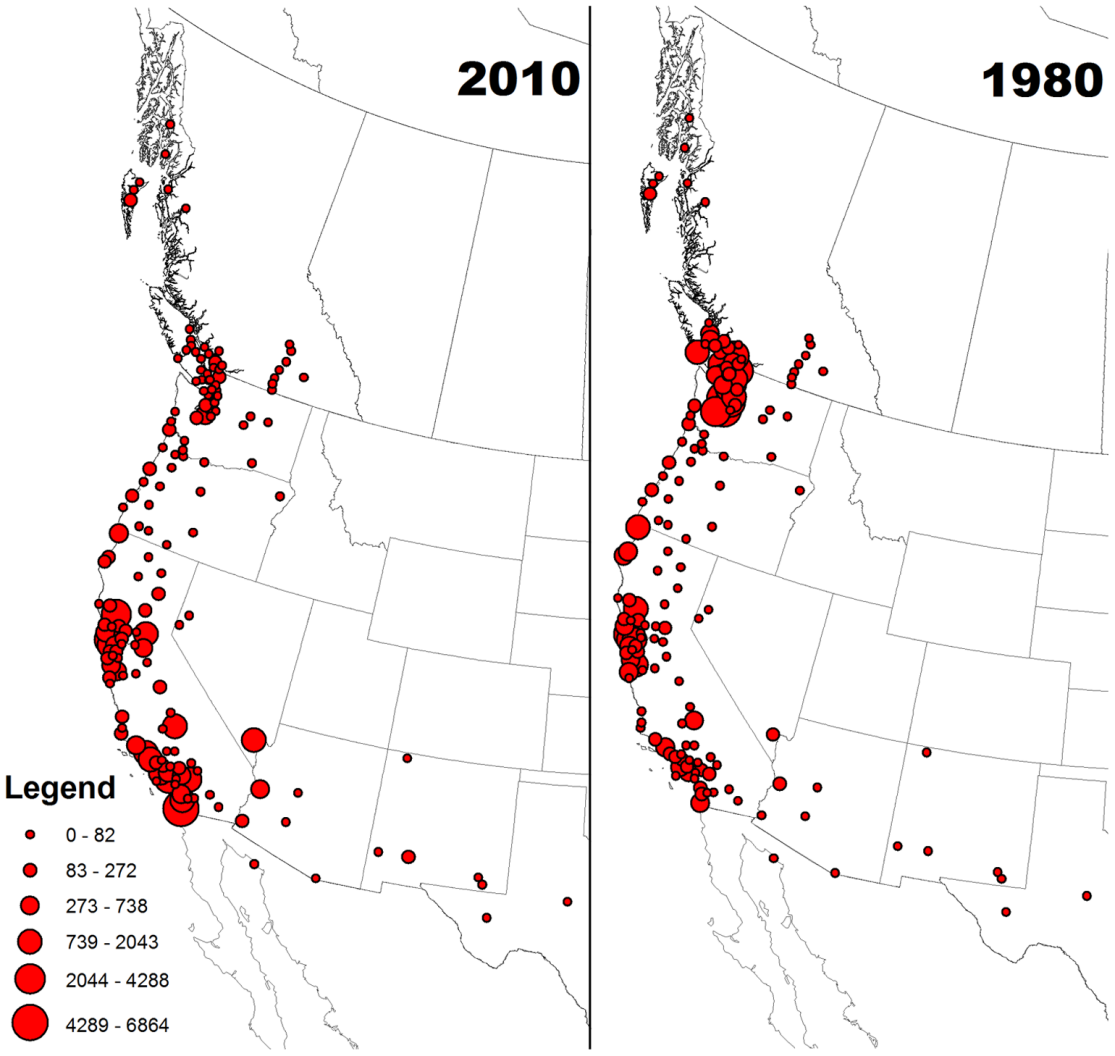
Distribution of non-breeding *Aechmophorus* grebes in 1980 and 2010. Each circle refers to an Audubon Christmas Bird Count used in the analysis and the size of the circle is scaled to the effort adjusted abundance of grebes on the count in that year. Combined data on western (*Aechmophorus occidentalis*) and Clark’s grebes (*A. clarkii*) were used in the figure but over 90% of observations are for western grebes in all regions except the southwestern states (see text for further detail).

We analyzed CBC surveys conducted from 1975–2010. The CBC began in 1900 and consists of a variable-effort survey based on a diameter 24.1 km count circle, conducted on a single day from mid-December to early January. Within each circle, observers record all species and individuals detected, the number of hours during which birds were surveyed, and mode of transport (e.g. foot, car, boat, etc.), facilitating estimates of survey effort during analysis [29]. We grouped 163 CBC circles into 8 regions based on historic distribution, latitude and location relative to the coast (Figure 1, Table 1). These regions represent all historic and current wintering concentrations, as well as less frequently used areas outside of the main range. Coastal and interior counts were identified for analysis because of distinct differences in prey communities. For coastal states and provinces, circles were designated as coastal if the center was <50 km from the coast or interior if greater >50 km from the coast based on the assumption that grebes made some use of estuaries, tidal rivers and marine waters even if detected on nearby inland waters. Most coastal counts (86 of 107) had centers <20 km from the coast. For consistency, we restricted our analyses to CBC circles wherein at least 10 grebes were detected in total and where surveys occurred in at least 50% of the years between 1975 and 2010. The CBC count circles above included ∼98% of all *Aechmophorus* grebe sightings in North America from 1975 to 2010. Analyses using a longer data set from 1970 produced identical results to those we report here but because of the increased CBC survey coverage after 1975 we focus on that time period.

**Table 1:** Location and number of CBC count circles and *Aechmophorus* spp. abundance in eight wintering regions.

Region	Description	CBC circles	Proportion WEGR	Mean count
Alaska/North BC	Coastal regions of southeast Alaska and British Columbia north of 51° latitude	7	100	97
Salish Sea^1^	Strait of Georgia, Puget Sound, Strait of Juan de Fuca and Vancouver Island	34	99.99	934
Outer Washington/Oregon Coast	Coastal counts along Washington and Oregon coasts	9	99.60	132
Northern California Coast	Coastal counts in California north of 36° latitude	26	93.05	660
Southern California Coast	Coastal counts in California south of 36° latitude including Baja California	25	96.54	661
Northern Interior	Interior counts in British Columbia, Washington and Oregon	25	99.35	11
Interior California/Nevada	Interior counts in California and Nevada	24	90.87	481
Southwestern Interior	Arizona, New Mexico, Texas and interior counts in Mexico	13	47.44	63

Coastal and interior circles are those <50 km or ≥50 km from the coast, respectively (see Methods). Mean count per circle is averaged across all 36 years and does not account for trend. Proportion WEGR refers to the percentage of all observations to species that were identified as western grebes (*Aechmophorus occidentalis*) since the split of western and Clark’s grebes in 1985.

1 The Salish Sea includes the inland network of waterways in southern British Columbia and northern Washington and includes the Strait of Georgia, Strait of Juan de Fuca, and Puget Sound.

### Statistical Analyses of Trend

Our first analysis focused on all *Aechmophorus* grebes between 1975 and 2010, while a second analysis examined trends in Clark’s grebes in the four southern regions from 1985 to 2010. Although the taxonomic split occurred in 1985, there may have been a lag in the response or ability of observers in distinguishing the two species. Thus, we compared trends for Clark’s grebe between two periods: the 24 years from 1987 to 2010 and 21 years from 1990 to 2010.

Count data were modeled as hierarchical over-dispersed Poisson variables and models were fit using Markov Chain Monte Carlo (MCMC) methods in WinBUGS 1.4.3 [30]. Each count *C_i,t_* at circle i in year t was assumed to follow a Poisson distribution with mean *µ_i,t_* and modeled as a log linear function of the explanatory variables, which include sampling effects, population effects and a noise component to account for over-dispersion. Noise was modeled as a random variable with mean 0 and survey specific variance (σ^2^_noise_). Effort is defined as the total number of survey hours per count including all modes of transportation. Because effort varies both spatially and temporally in CBC counts, we followed Link and Sauer [5] to estimate the effect of effort (Ψ) as:
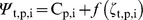
where C_p,i_ is the influence of circle i in region p, ζ_t,p,i_ represents the effort expended in producing the count on circle i in region p for year t, and *f*(ζ) is a function describing the influence of effort (total survey hours) on the number of birds counted. This latter influence is further described as:



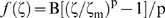
where ζ_m_ is the mean value of ζ_t,p,i_ and, B and p are parameters whose values accommodate a range of relationships between effort and the number of birds counted (see [5] for examples). B and p were initially allowed to differ by region but we found no support for geographic variation in the relationship and therefore a single relationship for all regions was assumed for the final model. The full count model, including the effort sub-model, was:



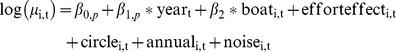



The base process model includes region-specific estimates for intercept (*β*_0_) and the time trend (*β*_1_), as well as a binary coefficient to account for whether or not a boat was used on a particular count (*β*_2_). We were unable to obtain specific hours for boat-based observations and therefore used a binary metric instead. All region-specific parameters were drawn from a common probability distribution with global mean and variance parameters. We assumed conditionally conjugate (vague) prior distributions for all parameters determining the µ_i,t_, for all x = 1,…,X covariate effects for all p = 1,…,P regions (for β_0_ and β_1_):
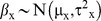



All µ_x_ were normally distributed with mean 0 and variance 10^3^. Effort in each circle and year was incorporated as described above. Additional variance components were included for the circle effect, the annual effect beyond trend, and noise. The precision parameters (τ^2^) for *β*_0–1_, B, circle, annual and noise were assigned uninformative inverse gamma prior distributions with shape and scale parameters equal to 0.001.

For each model we ran two Markov chains and examined convergence through individual parameter histories, Gelman-Rubin diagnostics and calculating MC error/sd [31]. Convergence was typically reached within 1,000 iterations but we used a higher burn-in period of at least 10,000 iterations before drawing samples from the posterior distribution. We used posterior predictive checks to examine the fit of the model to the data [31], [32]. For this method, a replicate data set is created at every iteration, allowing for a comparison of the actual data with replicated data that conform to model assumptions and are based on the parameters that created the model. A discrepancy measure, based on the sums of squared error, is used to compare the actual and replicated data sets under the premise that for a well fit model, about half of the points will lie above the 1∶1 line and half below it. Quantitatively, this can be expressed as a Bayesian p-value where values of the discrepancy measure close to 0 or 1 indicate a poor fit while values close to 0.5 indicate a good fit. In our case, the Bayesian p-value was 0.39 indicating a well fit model. We also conducted visual observations of the posterior distribution for the actual and replicated data to further examine model fit.

We used 95% credible intervals (CIs) generated from the posterior distribution of parameter estimates and interpreted significant trends as those where the 95% interval did not overlap zero. Region specific estimates of abundance were calculated by exponentiating the predicted count (*µ*_i,t_) for each circle and year. This approach allows for the inclusion of missing data for a count in a particular year and estimates that count as a derived statistic using all of the information in the model. Model corrected counts for all circles within a region were then used to produce a yearly regional index of abundance with 95% intervals. Multiple methods can be used to estimate trend over time. We used the geometric mean of proportional changes in population size between 1975 and 2010 expressed as a percentage [8]. Trend results using this approach were very similar to those using the log-linear trend coefficient over the full time series.

To look at the extent of a distribution shift over a 30 year period, we examined the mean center of occurrence in 1980 and 2010. The model corrected count for each circle and year were first obtained from the above analysis. We then used these counts to estimate the mean centre of occurrence for the two years using the following equation [3]:
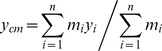
where y_cm_ is the latitude for the center of mass for the 163 CBC circles with mass (i.e. count) m_1_,m_2_,…m_n_ and latitudes y_1_,y_2_,_…._y_n._ WinBUGS code is provided in Methods S1.

## Results

Our analysis included 36 years of survey data from 163 CBC circles, representing 2,478,449 *Aechmophorus* grebe observations. Of all circles, 50 (30.7%) had surveys completed in all 36 years, 54 (33.1%) lacked surveys in 1 to 5 years, 28 (15.3%) lacked surveys in 6 to 10 years and 31 (19.0%) lacked surveys in 11 to 18 years. With the exception of northern BC and Alaska, the survey coverage increased similarly in all regions. Between the first and last ten years of this study, the addition of new circles ranged from a 1.14 fold increase in northern California to a 1.71 fold increase in the southwestern states. There was a greater increase from 2 to 7 circles in northern BC and southern Alaska. After 1985, grebes were recorded to species on 82% of counts (i.e. no individuals recorded as *Aechmophorus spp.*), with western and Clark’s grebes representing 98% and 2% of observations, respectively. The percentage of birds identified as western grebes varied by region but was very close to 100% in the portion of the winter range situated north of 42 degrees latitude, varied from 95–97% in coastal California, 92% in interior California and Nevada, and 47% in New Mexico, Arizona, Texas and interior Mexico (Table 1).

**Figure 2:**
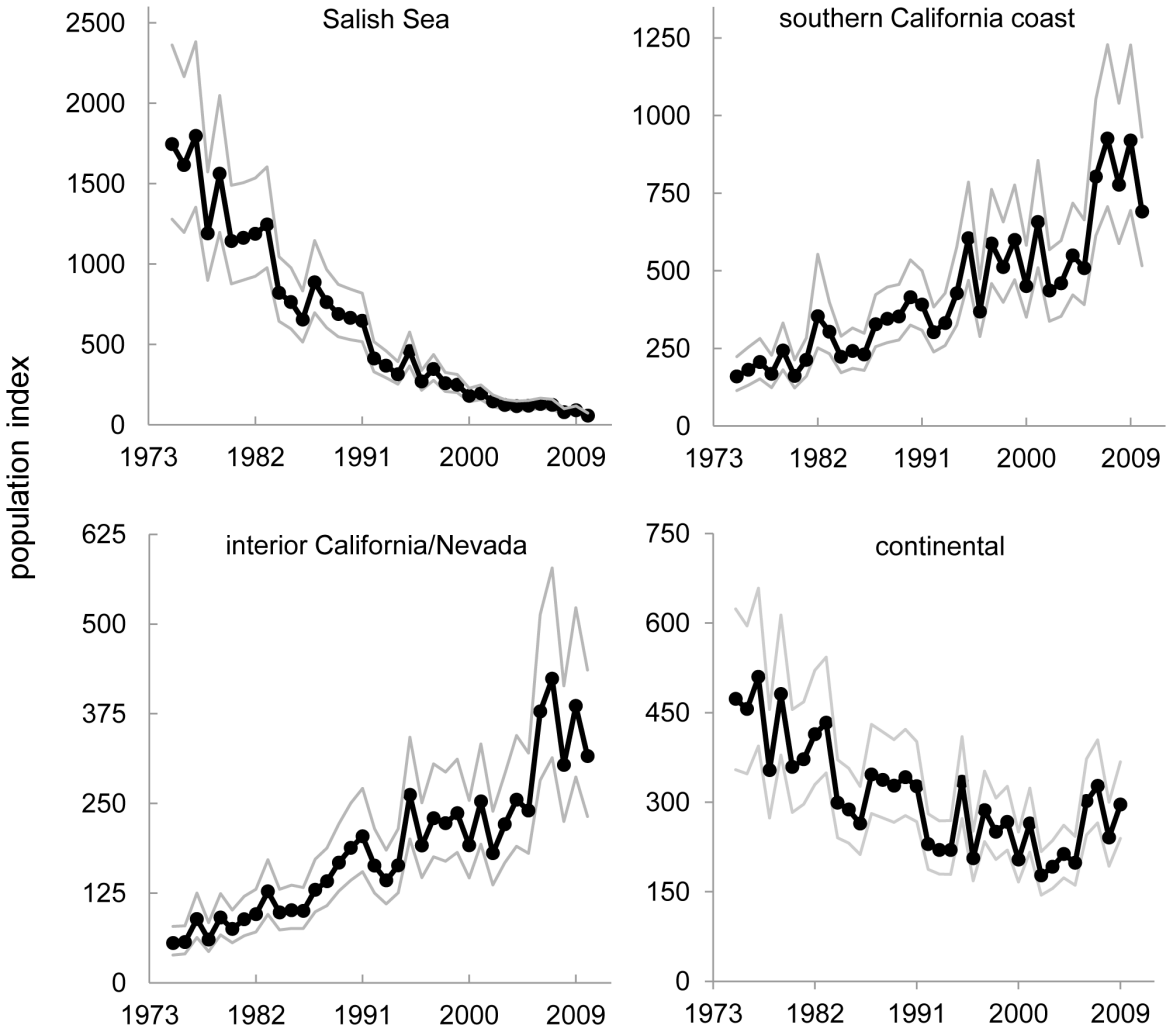
Population change of *Aechmophorus* grebes for the Salish Sea, southern California coast, interior California/Nevada and the continental population from 1975–2010. Population index is the predicted mean count per circle in each region controlled for survey effort. Light gray lines represent the 95% credible intervals on the mean count. Regions not shown here displayed no significant change with the exception of the southwestern states. Western grebes comprised >99% of the *Aechmophorus* spp. observations in the Salish Sea and 91–97% in the other two regions.

The continental population of *Aechmophorus* grebes declined at an annual rate of −2.07% (95% CI: −3.03, −1.11) resulting in a 52% decline between 1975 and 2010 (Figure 2). We also observed strongly contrasting trends across regions, especially in northern and southern parts of the range. Abundance in the Salish Sea declined strongly at −9.29%/yr (95% CI: −10.31, −8.26) and showed particularly steep declines after the late 1980s (Figure 2). In contrast, we observed strong increases in abundance in the southern regions, including coastal Southern California and Northern Mexico (4.30%/yr, 95% CI: 3.00, 5.60), interior California and Nevada (5.10%/yr, 95% CI: 3.90, 6.40) and the southwestern interior states (6.70%/yr, 95% CI: 4.80, 8.60). We observed no significant change in abundance in coastal Alaska and Northern BC (0.30%/yr, 95% CI: −2.41, 3.10), the outer Washington and Oregon coasts (−0.12%/yr, 95% CI: −1.87, 1.60), coastal Northern California (−0.73%/yr, 95% CI: −1.88, 0.40) or the northern interior (0.60%/yr, 95% CI: −0.77, 2.00). The largest component of variance in the overall model was related to differences in the average abundance among regions (posterior standard deviation (σ) = 2.44, 41.9% of variance), followed by differences in the average abundance among circles within regions (σ = 1.87, 32.0%). The use of boats had no significant effects on the number of grebes recorded on a count (β_boat_ = 0.031, 95% CI: −0.115, 0.173).

Similar patterns were apparent in analyses to estimate the center of occurrence for the wintering distribution in 1980 and 2010. In 1980, the mean center of occurrence was estimated at 44.65 degrees latitude. By 2010 the estimated mean center of occurrence was 36.73 degrees latitude, which represents a southward shift of approximately 895 km over 30 years.

The average number of Clark’s grebes recorded in the 4 southern regions approximately tripled in the first few years of the study from an average of 7.0 individuals per count (95% CI: 4.9, 10.0) in 1987 to 21.2 (95% CI: 15.4, 29.8) in 1990. This increase resulted in a significant annual change of 6.00%/yr between 1987 and 2010 (95% CI: 3.90, 8.10), despite little or no increase between 1990 and 2010 (1.20%/yr, 95% CI: −1.17, 3.50), perhaps suggesting that CBC volunteers more often identified Clark’s grebes as western grebes early on. From 1990 to 2010, Clark’s grebes increased significantly in the southwestern states at 6.60%/yr (95% CI: 2.30, 11.30), but not in any other region (Northern Coastal California (−1.07, 95% CI: −3.55, 1.50), Southern Coastal California (0.10, 95% CI: −2.59, 2.80), Interior California and Nevada (2.70, 95% CI: −0.12, 5.60)).

## Discussion

The non-breeding range of western grebes has undergone a marked southward shift of nearly 900 km during the past three decades. Population abundance has declined by over 95% in the Salish Sea, which represented the core of the winter distribution in the late-1970s, but increased steadily by over 300% in southern coastal and interior California. Over the same period, and accompanying this dramatic regional shift, we estimated a 52% decline in the size of the North American population. We first discuss hypotheses to explain the southward range shift and studies that could test among them, and then examine the continental decline in further detail.

### Potential Mechanisms Underlying a Distribution Shift

An extensive shift in the species non-breeding distribution suggests a change in the favorability of environmental conditions across the winter range. Western grebes are a mobile species that do not display strong fidelity to particular wintering areas and banding records show that individuals move along the coast within the same season [33], [34]. Thus, large-scale movements of this species over time might be expected as birds track conditions on the wintering grounds that affect individual fitness, such as resource availability [35].

Western grebes are almost entirely piscivorous [27], [36] and in winter, primarily select fish in the 80–100 mm size range as prey [37]. Thus, a more specific hypothesis is that the range shift we observed is a consequence of changes in the abundance and distribution of their prey. Shifts in the availability of forage fish on the distribution of marine birds have been noted previously for cape gannets (*Morus capensis*) in the Benguela current ecosystem off southern Africa [38] and for Balearic shearwaters (*Puffinus mauretanicus*) in the northeast Atlantic [25]. Limited evidence suggests that western grebes use a variety of fish species as prey on the Pacific coast, but also that a small number of species probably represent a very large fraction of all prey biomass available to them, particularly Pacific sardine (*Sardinops sagax*), Pacific herring (*Clupea pallasii*) and northern anchovy (*Engraulis mordax*). Since the mid-1980s, Pacific sardine have increased rapidly in coastal California and northern Mexico, linked to an oceanic regime shift favoring this species, as well as a recovery from extensive overfishing in the early half of the 20^th^ century [19], [39], [40]. The biomass of the Pacific sardine stock was estimated at just a few thousand metric tonnes in 1985, but nearly 2 million metric tonnes by 2000, potentially representing a massive increase in prey for wintering grebes. Recent acoustic trawl surveys in the California Current system indicate that Pacific sardine in coastal California represent the majority of the forage fish biomass. In contrast, the biomass further north along the Washington coast is far lower [41].

Similarly, the range shift we observed may also be related to regional declines in the quantity or availability of Pacific herring, which have been identified as a preferred prey of western grebes in the Salish Sea [37]. Several formerly large herring stocks in northern Puget Sound crashed after 1990, and currently fewer than half of Puget Sound herring stocks are considered to be ‘healthy’ or ‘moderately healthy’ [42]. Adult herring abundance in the Strait of Georgia and on the west coast of Vancouver Island has also fluctuated since the 1970s, but is currently at or below average stock sizes observed from about 1960 to present [43]. Moreover, there is evidence of a pronounced reduction in the frequency of early and late season spawning by Pacific herring in the Salish Sea (January/early February and April/May respectively), a long-term reduction in size-at-age [44], [45] and a reduction in the extent and number of spawning locations [45].

Fluctuations in the abundance of northern anchovy and other potential prey not harvested commercially (e.g. Pacific sand lance *Ammodytes hexapterus*) are poorly known and require further study. According to [41], northern anchovy are uncommon when compared to Pacific sardine at present, but both species vary in abundance within regions as oceanic conditions change [19], and it is therefore unclear how important anchovy may be to the diet of western grebes. Stable isotope analyses are currently underway to describe long-term trends in diet composition in western grebes wintering in the Salish Sea and coastal California and to test for links between diet and forage fish abundance. There is also a need for consideration of other changes in oceanic conditions that might affect grebes including anthropogenic disturbance related to marine traffic and changes in predation pressure related to recovering bald eagle (*Haliaeetus leucocephalus*) populations [46], which may interact with resource abundance to influence the quality of winter habitat for western grebes.

### Continental Declines and Potential Causes

Our estimate of continental-scale declines was based on data from 163 CBC circles covering the vast majority of the *Aechmophorus* grebe winter range. The estimate of a 52% decline relies on the assumption that the CBC data we used sampled the wintering population randomly, which may be false if *Aechmophorus* grebes that formerly overwintered in areas included in CBC surveys now overwinter in large numbers in unsurveyed regions of the Pacific coast. This assumption appears sound over the range from Alaska through Southern California, because the number of Pacific coast counts north of Mexico has increased similarly in all regions since 1975, and because *Aechmophorus* grebes prefer sheltered waters such as the Salish Sea and San Francisco Bay over those on the exposed outer coast [27]. Sheltered waters of the United States and southern British Columbia are relatively well surveyed by the CBC. In contrast, if western and Clark’s grebes now overwinter in larger numbers in coastal Baja California, an area with few CBC surveys, then our estimate for the continent-wide decline may overestimate the true decline. The three surveys that are available for Baja California suggest lower numbers in this region compared to coastal Southern California (mean effort adjusted grebes per survey = 223, 22 and 4), but it is difficult to draw conclusions with small sample sizes and additional surveys in Baja California will be needed to test this possibility in greater detail. Because Clark’s grebe abundance remained about stable from 1990 to 2010, the continent-wide decline that we detected in western grebes suggests that different factors may be operating to influence winter abundance and distribution in these species. Breeding ground trends based on the Breeding Bird Survey indicate a non-significant survey wide decline of −1.0% annually (95% CI: −6.2%, 2.1%) for western and Clark’s grebe combined [47]. However, due to their colonial breeding habits and selection of larger lakes, roadside surveys are not a reliable monitoring technique for these species.

Several hypotheses might explain range-wide declines in western grebes together or singly. Breeding grebes require lakes large enough to support sufficient fish prey, as well as sheltered marshes with emergent vegetation for nesting, and protection from high water and wave action [27], [48], [49]. The loss and degradation of breeding habitat via wetland draining and water management is well documented [27], [50]. Lakes suitable for grebes are also often used for recreation, which can degrade nesting habitat via shoreline development [49]. Long-term observations on breeding abundance are patchy for western grebes, but several observations are consistent with the hypothesis of larger scale declines in breeding habitat quality. First, the number of nesting colonies in Alberta, which supported up to 20% of the continental population in the 1970s, have decreased in recent decades along with a decline in the provincial population [26], [51]. Some breeding colonies in British Columbia also declined or disappeared after 1970 [52]. In northern California, human disturbance and pollution are both recognized as threats to breeding western and Clark’s grebes [53].

Western grebes also face various threats in winter, when their preference for near-shore habitats leads to frequent oiling [16], [34] and incidental capture in gill nets [15]. However, no studies have quantified the sources of winter mortality or estimated the relative contribution of events during the breeding and non-breeding seasons to regional population dynamics. Additional research is therefore needed in breeding areas to test if the apparent continental decline we have detected represents a shared decline across the range or is due to severe declines in particular regions.

In summary, we have shown how the combined application of citizen science data at range-wide scales and hierarchical Bayesian approaches provided insight into the potential causes of localized population declines for a migratory waterbird. Our results suggest that western grebe populations have declined at the continental scale since 1975, but perhaps more importantly, we have demonstrated a dramatic southward range shift that we suggest may be related to large-scale fluctuations in the spatial distribution and abundance of their forage fish prey base. However, in the absence of detailed data on spatial and temporal variation in forage fish prey, drawing clear statistical links between correlated trends in grebe and prey abundance over time is difficult. To remedy this situation and identify the causal factors underlying grebe abundance and distribution patterns will require additional studies to track individual animals between breeding and wintering habitats across years and compare historic and current diets of western grebes.

## Supporting Information

Methods S1 **WINBUGS Code used to estimate regional non-breeding population trends of** ***Aechmophorus*** **grebes from 1975 to 2010 using Audubon Christmas Bird Count data.**
(DOCX)
