# Supplementary material for: Citizen Science Reveals an Extensive Shift in the Winter Distribution of Migratory Western Grebes
Source: PLoS One. 2013 Jun 19;8(6):e65408. doi: 10.1371/journal.pone.0065408 (PMC3686804; doi:10.1371/journal.pone.0065408)
Supplement: Methods S1 — WINBUGS Code used to estimate regional non-breeding population trends of Aechmophorus grebes from 1975 to 2010 using Audubon Christmas Bird Count data. (DOCX) [file pone.0065408.s001.docx]

Methods S1.

Model

{

for(i in 1:ncircles)

{

for (t in 1:nyears)

{

mu.loglambda[i,t] <- beta0[pop[i,t]] + beta1[pop[i,t]]*yr[t] + beta2*boat[i,t] + circ[circle[i,t]] + efforteffect[i,t] + annual[ann[i,t]]

loglambda[i,t]~dnorm(mu.loglambda[i,t],taunoise)

log(lambda[i,t])<-loglambda[i,t]

count[i,t] ~ dpois(lambda[i,t])

countnew[i,t]~dpois(lambda[i,t])

err[i,t]<-pow(count[i,t]-lambda[i,t],2)/lambda[i,t]

newerr[i,t]<-pow(countnew[i,t]-lambda[i,t],2)/lambda[i,t]

predcount[i,t]<-exp(mu.loglambda[i,t])

}

}

fit<- sum(err[,])

fitnew <- sum(newerr[,])

test<-step(fitnew-fit)

bpvalue <- mean(test)

for (t in 1:nyears)

{

p1[t]<-mean(predcount[1:7,t])

p2[t]<-mean(predcount[8:41,t])

p3[t]<-mean(predcount[42:50,t])

p4[t]<-mean(predcount[51:75,t])

p5[t]<-mean(predcount[76:97,t])

p6[t]<-mean(predcount[98:122,t])

p7[t]<-mean(predcount[123:150,t])

p8[t]<-mean(predcount[151:163,t])

ptot[t]<-mean(predcount[1:163,t])

}

p1c<-pow((p1[36]/p1[1]),0.02857)

p2c<-pow((p2[36]/p2[1]),0.02857)

p3c<-pow((p3[36]/p3[1]),0.02857)

p4c<-pow((p4[36]/p4[1]),0.02857)

p5c<-pow((p5[36]/p5[1]),0.02857)

p6c<-pow((p6[36]/p6[1]),0.02857)

p7c<-pow((p7[36]/p7[1]),0.02857)

p8c<-pow((p8[36]/p8[1]),0.02857)

ptotc<-pow((ptot[36]/ptot[1]),0.02857)

for(r in 1:npops)

{

beta0[r]~ dnorm(mubeta0, taubeta0)

beta1[r]~ dnorm(mubeta1, taubeta1)

}

beta2~dnorm(0,0.001)

mubeta0~ dnorm(0, 0.001)

mubeta1~ dnorm(0, 0.001)

taubeta0 ~dgamma(.001,.001)

sigbeta0<-1/sqrt(taubeta0)

taubeta1 ~dgamma(.001,.001)

sigbeta1<-1/sqrt(taubeta1)

taunoise ~dgamma(.001,.001)

signoise<-1/sqrt(taunoise)

# circle effect

for (c in 1:ncircles)

{

circ[c]~dnorm(0, taucirc)

}

taucirc ~ dgamma(.001,.001)

varcirc<-1/taucirc

sigcirc<-1/sqrt(taucirc)

#annual effect

for (a in 1:nann)

{

annual[a]~dnorm(0, tauann)

}

tauann ~ dgamma(.001,.001)

varann<-1/tauann

sigann<-1/sqrt(tauann)

# Effort Effect

for(i in 1:ncircles)

{

for (t in 1:nyears)

{

scaledeffort[i,t]<-effort[i,t]/108.2

trans.effort[i,t]<- (pow(scaledeffort[i,t],p)-1)/p

efforteffect[i,t] <- B*trans.effort[i,t]

}

}

p~dunif(-4,4)

B~dnorm(0.0,0.0001)

}
